# Downsizing a long-term precipitation network: Using a quantitative approach to inform difficult decisions

**DOI:** 10.1371/journal.pone.0195966

**Published:** 2018-05-07

**Authors:** Mark B. Green, John L. Campbell, Ruth D. Yanai, Scott W. Bailey, Amey S. Bailey, Nicholas Grant, Ian Halm, Eric P. Kelsey, Lindsey E. Rustad

**Affiliations:** 1 Center for the Environment, Plymouth State University, Plymouth, NH, United States of America; 2 Northern Research Station, USDA Forest Service, Durham, NH, United States of America; 3 Department of Forest and Natural Resources Management, SUNY College of Environmental Science and Forestry, Syracuse, NY, United States of America; 4 Department of Atmospheric Science and Chemistry, Plymouth State University, Plymouth, NH, United States of America; 5 Mount Washington Observatory, North Conway, NH, United States of America; Oregon State University, UNITED STATES

## Abstract

The design of a precipitation monitoring network must balance the demand for accurate estimates with the resources needed to build and maintain the network. If there are changes in the objectives of the monitoring or the availability of resources, network designs should be adjusted. At the Hubbard Brook Experimental Forest in New Hampshire, USA, precipitation has been monitored with a network established in 1955 that has grown to 23 gauges distributed across nine small catchments. This high sampling intensity allowed us to simulate reduced sampling schemes and thereby evaluate the effect of decommissioning gauges on the quality of precipitation estimates. We considered all possible scenarios of sampling intensity for the catchments on the south-facing slope (2047 combinations) and the north-facing slope (4095 combinations), from the current scenario with 11 or 12 gauges to only 1 gauge remaining. Gauge scenarios differed by as much as 6.0% from the best estimate (based on all the gauges), depending on the catchment, but 95% of the scenarios gave estimates within 2% of the long-term average annual precipitation. The insensitivity of precipitation estimates and the catchment fluxes that depend on them under many reduced monitoring scenarios allowed us to base our reduction decision on other factors such as technician safety, the time required for monitoring, and co-location with other hydrometeorological measurements (snow, air temperature). At Hubbard Brook, precipitation gauges could be reduced from 23 to 10 with a change of <2% in the long-term precipitation estimates. The decision-making approach illustrated in this case study is applicable to the redesign of monitoring networks when reduction of effort seems warranted.

## Introduction

Environmental monitoring networks have contributed to important scientific advances and have been essential for shaping environmental policy [1]. Effective monitoring involves collecting the amount and quality of data necessary to meet objectives [2]. Despite the crucial role of these networks, funding for monitoring programs can wax and wane in response to the interests and resources of investigators, supporting institutions, and funding agencies [3]. As budgets and research needs change over time, it is essential to periodically reevaluate the scope of monitoring to ensure that programs continue to match needs [4]. While consistency of methods is integral to long-term monitoring, flexibility is also important to success. Adaptive monitoring programs evolve iteratively as research questions change and new information becomes available [5].

Measuring precipitation is important for evaluating aspects of the environment such as air quality, water supply, and the risk of flood and drought. The precipitation monitoring program at the Hubbard Brook Experimental Forest in New Hampshire, USA, is used to track annual water budgets of nine headwater catchments to detect change over time [6] and the impact of forest manipulations [7,8]. Precipitation data from the site are also used to calculate catchment-scale element budgets (e.g., calcium [9]) which have been important for assessing impacts on forest biogeochemistry of legislation such as the Clean Air Act [10,11].

Because precipitation can be strongly influenced by local topography and storm type, it has high spatial variability and requires a distributed network of gauges for measurement [12–15]. One of the challenges in establishing precipitation gauge networks is determining the appropriate number and placement of gauges across the landscape for an acceptable level of confidence [16–18]. Improvements in precipitation monitoring efficiency can enhance monitoring programs by directing appropriate reallocation of scarce resources. While optimization of sampling is essential for ensuring that monitoring designs are cost-effective, examples of how to balance logistical constraints and high quality data are limited [19–23]. Methods for evaluating monitoring programs become increasingly important as funding for environmental monitoring declines.

Our goal in this study was to use a quantitative uncertainty analysis to make decisions about downsizing a precipitation network at the Hubbard Brook Experimental Forest in New Hampshire, USA. Precipitation has been monitored with a network of 23 gauges distributed across nine small catchments beginning in 1955. We simulated reductions in sampling intensity, with the intent of developing a reduced sampling scheme that would produce estimates of precipitation volume at the catchment scale that are as close as possible to the values obtained with the current intensive monitoring scheme. We evaluated the sensitivity of precipitation estimates to the removal of individual gauges, and we considered every possible number and combination of gauges to retain. Because calculating precipitation inputs to the catchments depends on interpolating precipitation volume, we repeated this analysis with two interpolation methods, Theissen polygons, the traditional method used at Hubbard Brook, and inverse distance weighting (IDW), an alternative method. We used these scenarios to choose a reduced precipitation network scenario that produced acceptable deviations from our current precipitation estimates given our logistical constraints. We then tested the impact of our gauge-reduction scenario on precipitation at daily, monthly, and annual time scales to ensure acceptable performance of the reduced network. Finally, we showed how historical estimates of three other variables that are dependent on precipitation measurements–annual evapotranspiration and bulk deposition of sulfate and calcium–would change in response to hypothetical reductions in precipitation monitoring. This process gave us confidence that the reduced network produced similar precipitation estimates and thus maintained the integrity of the long-term data.

## Methods

### Site description and data collection

The Hubbard Brook Experimental Forest was established in 1955 by the USDA Forest Service as a center for hydrologic research in the White Mountains of New Hampshire, USA (43°56′N, 71°45′W). The topography was carved by continental glaciation and is characterized by hilly to steep mountainous terrain. The climate is humid continental, with average monthly air temperatures ranging from -9°C in January to 19°C in July. Average annual precipitation ranges from 1421 to 1626 mm across the gauges (Table 1) and is distributed fairly evenly throughout the year. Approximately 30% of precipitation falls as snow, and a snowpack usually persists from late December until mid-April. Heavy precipitation events (≥50 mm per day, averaged across all gauges) occur most frequently from August to October [24]. Most of these events are predominantly stratiform but 21% are convective and thus have high spatial heterogeneity [24].

**Table 1:** Location, elevation, and date of establishment of precipitation gauges at the Hubbard Brook Experimental Forest, New Hampshire, USA. Volume is the average annual precipitation from 1998 to 2014. Note that RG18 was decommissioned in 1975. RG22 is not included in this analysis because it is located far from the gauged catchments (at the U.S. Forest Service headquarters). Thus there are 23 precipitation gauges in this study.

Gauge	Latitude (dd)	Longitude (dd)	Elevation (m)	Established	Volume (mm) (SD)
RG1	43.9521	-71.7248	490	1 Jan 1956	1464 (181)
RG2	43.9550	-71.7270	560	1 Jan 1956	1498 (190)
RG3	43.9587	-71.7283	715	1 Jan 1956	1421 (167)
RG4	43.9552	-71.7208	565	1 Aug 1956	1448 (169)
RG5	43.9593	-71.7183	670	1 Nov 1957	1456 (171)
RG6	43.9572	-71.7349	745	1 Jan 1960	1496 (183)
RG7	43.9536	-71.7332	605	1 Jan 1960	1484 (178)
RG8	43.9512	-71.7288	510	1 Jan 1960	1484 (179)
RG9	43.9557	-71.7418	760	1 Dec 1963	1508 (192)
RG10	43.9536	-71.7399	670	1 Dec 1963	1540 (192)
RG11	43.9502	-71.7346	550	1 Dec 1963	1488 (181)
RG12	43.9276	-71.7662	620	1 Jan 1965	1534 (201)
RG13	43.9205	-71.7692	765	1 Jan 1965	1545 (202)
RG14	43.9208	-71.7656	730	1 Jan 1965	1567 (218)
RG15	43.9233	-71.7610	770	1 Jan 1965	1548 (206)
RG16	43.9165	-71.7668	860	1 Jan 1965	1559 (200)
RG17	43.9190	-71.7582	895	1 Jan 1965	1535 (201)
RG19	43.9293	-71.7594	595	1 Nov 1968	1544 (210)
RG20	43.9240	-71.7575	790	1 Sep 1968	1540 (205)
RG21	43.9225	-71.7526	800	1 Sep 1968	1568 (205)
RG23	43.9272	-71.7469	665	1 Jan 1996	1559 (197)
RG24	43.9197	-71.7504	790	5 Jan 1997	1626 (214)
RG25	43.9222	-71.7429	810	5 Jan 1997	1521 (194)

**Fig 1:**
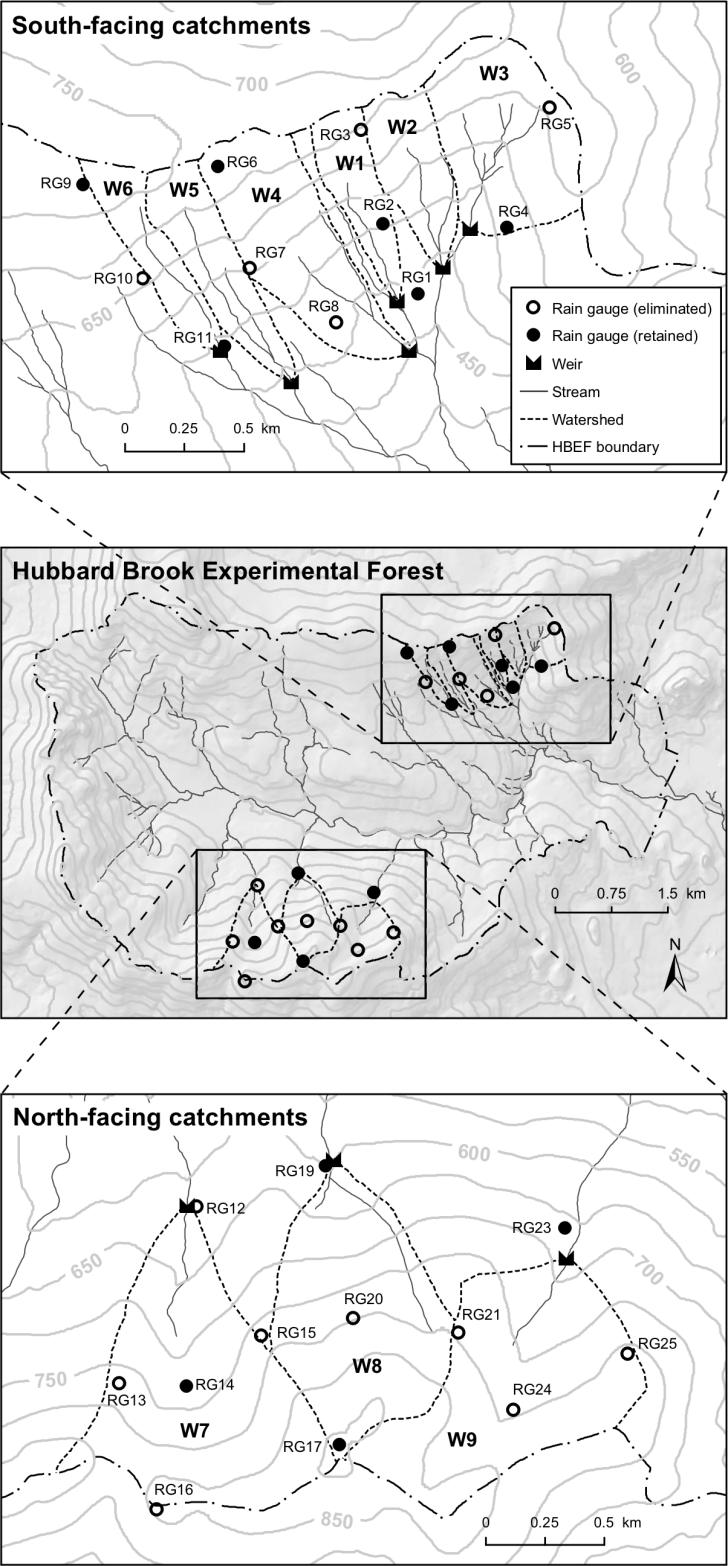
Map of precipitation gauges (designated RG1 through RG25, excluding RG18 and RG 22). Rain gauges were located to best characterize precipitation inputs to small headwater catchments (designated W1 through W9) monitored for streamflow at the at the Hubbard Brook Experimental Forest.

Research at Hubbard Brook has focused on the hydrology and biogeochemistry of experimental manipulations and long-term patterns in nine small catchments [25]. Gauged catchments range in size from 12 to 42 ha on the south-facing slope of the Hubbard Brook valley, with larger, 59 to 77 ha, catchments on the north-facing slope (Fig 1). Catchment-scale precipitation inputs have been monitored with 11 gauges on the south-facing slope and 12 gauges on the north-facing slope. A total of 25 gauges have been installed at Hubbard Brook, numbered chronologically in order of establishment. Data from RG18 was omitted from this analysis because it was discontinued in 1975; it was added in the first catchment to be experimentally deforested but was removed because it recorded similar volumes as the gauges around it. Data from RG22 was not included because it is located at the U.S. Forest Service headquarters building, away from the area of the experimental catchments.

Precipitation volume is measured weekly with National Weather Service 8-inch (20-cm) standard precipitation gauges. The gauges are mounted so that the orifice is ~2–3 m above the ground, which ensures that it remains well above the deepest snowpack. Single Alter wind shields are installed to reduce wind-induced catch error [26]. All gauges are located in clearings that are maintained free of vegetation within a 40° angle of the gauge. The trees are approximately 20 m tall [27]. Weight-recording gauges are co-located with standard precipitation gauges at six locations (RG1, 6, 10, 14, 19, 23). Daily precipitation values for each standard gauge are obtained by prorating the weekly totals using daily totals from the nearest weight-recording gauge [28].

### Data analysis

Our data analysis focused on catchment-scale precipitation estimates, which are generated with multiple precipitation gauges. The best daily, monthly, and annual precipitation estimates were those based on the full 23-gauge network [29]. We simulated network reductions, recalculated catchment-scale precipitation, and evaluated the deviations resulting from these simulated network reductions relative to the full network.

We quantified the most influential gauges for individual catchments by omitting each gauge individually, recalculating the spatial interpolation, and then calculating the long-term average precipitation estimate for each catchment.

We considered all possible scenarios of sampling intensity for the south-facing catchments (2047 combinations) and the north-facing catchments (4095 combinations), from the current scenario, with 11 or 12 gauges, to only 1 gauge remaining. The precipitation inputs to each catchment were calculated for each scenario. This analysis was based on the 1998 to 2012 average precipitation volume.

Calculating precipitation inputs at the catchment scale requires choosing a method for interpolating among gauges. Since the inception of the Hubbard Brook study in 1955, precipitation input to each of the experimental catchments has been estimated using a Thiessen polygon spatial weighting scheme [30], in which each point in the landscape is described by the nearest gauge. We included this approach in some of our analyses, but used inverse distance weighting (IDW) as the primary method for spatial interpolation. The IDW method is similar to the Thiessen polygon method in that it assigns values to each point on the landscape based on the nearest neighbors, but the interpolation is smooth and thus less obviously unrealistic.

Other interpolation models were considered but not used in this study. Linear regression was not appropriate because there were not strong relationships between precipitation and potential explanatory variables such as elevation or longitude [24]. Relationships do occur at this site with latitude and longitude for individual events depending on the low-level wind field, but these individual event patterns are not seen over seasonal or longer time scales [24]. Kriging did not provide useful information because the gauges are quite evenly distributed across each group of catchments, and thus the semivariogram does not describe covariance at smaller scales. Finally, fixed spatial weighting schemes, such as IDW and Theissen polygons, are computationally attractive because they do not require recalculation at each time step, unlike kriging, for example.

We selected a reduced sampling scheme for further analysis. For the south-facing slope, we selected RG1, 2, 4, 6, 9, and 11; for the north-facing slope, we selected RG14, 17, 19 and 23. These emerged as the favored scenario, as will be presented below. To evaluate the effect of a reduced sampling scheme on precipitation estimates at various time scales, we compared daily, monthly, and annual precipitation estimates on the south-facing slope for the 6-gauge network to the full set of 11 gauges. We used only the south-facing catchments, instead of the north-facing catchments, because they have been instrumented longer and thus provide more years of data for this analysis (1964 to 2014). Comparisons of daily estimated precipitation were calculated only for days where at least one of the south-facing gauges detected precipitation. Cumulative frequency distributions were used to compare the differences between precipitation volumes estimated by the reduced and full gauge sets at daily, monthly, and annual time scales.

To test the impact of this new, reduced gauge scenario on other calculations that rely on precipitation estimates, we recalculated evapotranspiration and bulk deposition of calcium and sulfate to W6, the biogeochemical reference catchment. Evapotranspiration is estimated annually as the difference between precipitation input and streamflow output, which is measured with weirs and flumes at the outlet of each catchment [31]. Bulk deposition of calcium and sulfate is estimated by collecting a cumulative weekly bulk atmospheric deposition sample, and multiplying the solute concentration in that sample by the weekly precipitation [9]. One bulk deposition collector, consisting of a polyethylene funnel connected to a polyethylene bottle, is co-located at RG11 [32].

For most of the analyses, we used data from Jan 1, 1998 to Dec 31, 2014, because the last of the gauges on the north-facing slopes was established in 1998. For the analysis of daily, monthly, and annual deviations, we used the full period for which data were available for Watershed 6 (1964 to 2014), and for computing evapotranspiration and solute fluxes at W6, the biogeochemical reference catchment.

All analyses were performed in the statistical computing language R [33]. The R package *deldir* [34] was used for the Thiessen polygon and *gstat* [35] for inverse distance weighting.

## Results

### Spatial pattern in precipitation volume

Accurate spatial characterization of precipitation volume requires a dense precipitation gauge network. In our data set, there are significant differences between the groups of gauges on the north and south face of the Hubbard Brook basin.

From 1998 to 2014, the gauges on the north-facing slopes received an average of 73 mm per year more precipitation than the gauges on the south-facing slopes (p<0.001 in a t-test). Average annual precipitation ranged from 1421 mm (RG3) to 1540 mm (RG10) on the south-facing slopes and from 1521 mm (RG25) to 1626 mm (RG24) on the north-facing slopes (Table 1; Fig 1).

The gauges on the north-facing slopes extend to higher elevation than those on the south-facing slopes. Across all 23 gauges, mean annual precipitation (1998 to 2014) showed a weak relationship with elevation in regression (r^2^ = 0.28; p = 0.01), but the relationship was not significant within the areas of the south-facing (r^2^ = 0.02; p = 0.72) or north-facing slope (r^2^ = 0.01; p = 0.81). For this reason, elevation is not a good candidate for a covariate in interpolating precipitation volume within either group.

The relationship with longitude was more significant than that with elevation (r^2^ = 0.61; p<0.001). On the south-facing slope, precipitation decreases from west to east (r^2^ = 0.53; p = 0.01), but this relationship is not significant on the north-facing slope (r^2^ = 0.15; p = 0.235). The apparent effect of longitude on precipitation in the south-facing slopes is due to RG10 being high precipitation and RG3 being low precipitation, as regression without these two gauges does not have a significant east-west gradient (r^2^ = 0.12; p = 0.28).

Latitude is also significant when analyzed across all the gauges (r^2^ = 0.67; p<0.001), as the north-facing slopes are south of the south-facing slopes. This relationship is not significant within either the south-facing group (r^2^ = 0.15; p = 0.24) or the north-facing group (r^2^ = 0.09 = 0.35).

Because of the lack of strong regression models for predicting landscape-scale precipitation, we used interpolation methods that are based only on distance (i.e., IDW and Theissen polygons).

### Seasonal pattern in precipitation volume

**Fig 2:**
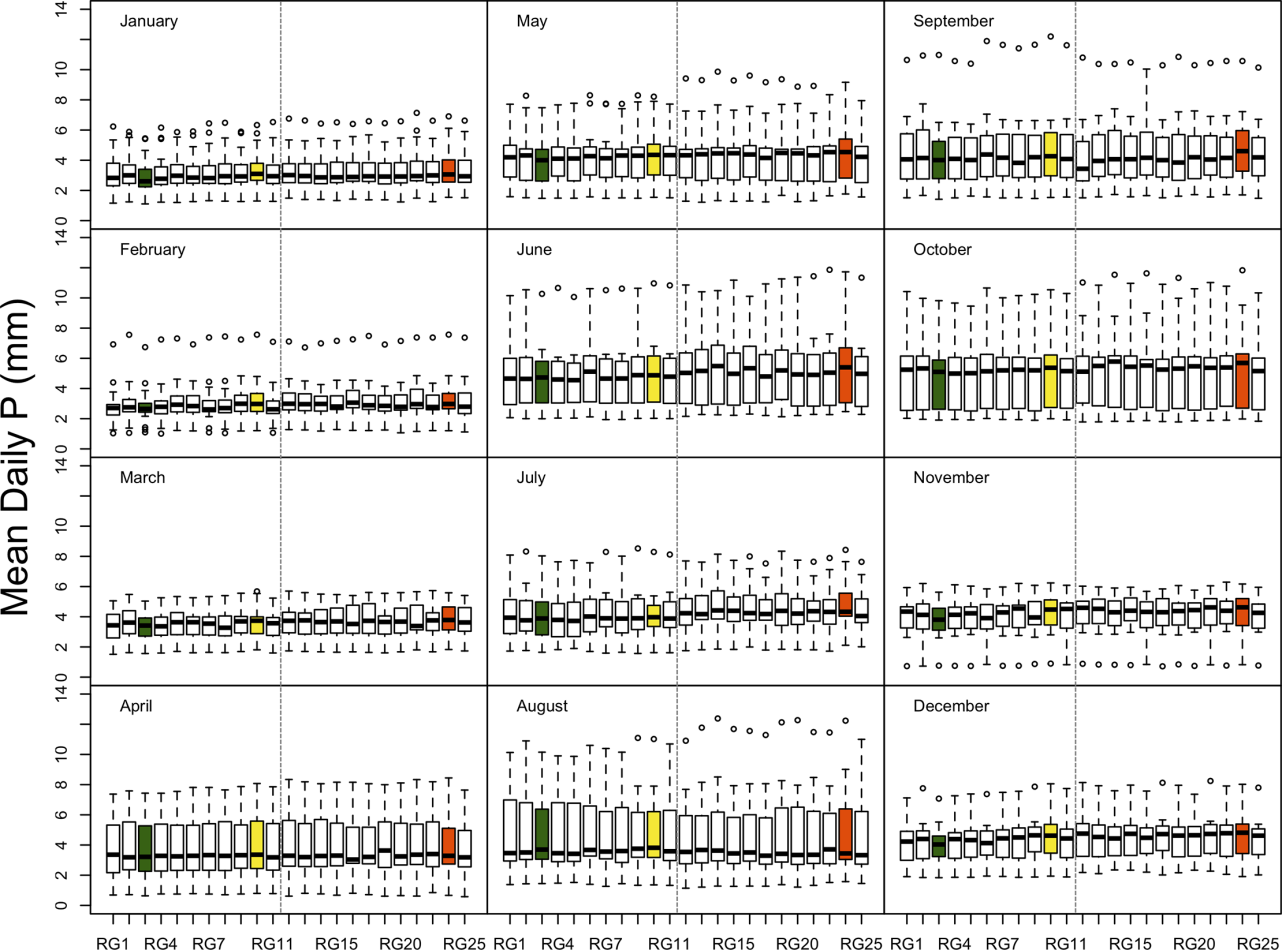
Boxplots of monthly precipitation (mm day^-1^) for the 1998 to 2014 period. Boxes extend from the 1^st^ to the 3^rd^ quartile and the line shows the median. The whisker extends up to 1.5 times the and interquarile range (IQR) from the box or to the most extreme value. Open circles indicate values more than 1.5 times the IQR from the box. Vertical dashed lines separate the north and south facing gauges. Colored boxes highlight gauges discussed in the text, with colors corresponding to Fig 6.

To understand the observed temporal patterns in precipitation, we inspected the monthly distribution of precipitation, by gauge, over the 17-year period of this analysis, using mean daily precipitation to account for differences in the number of days per month (Fig 2). Although precipitation amount is not strongly seasonal in this climate, January to April tends to have consistently low values. August and October are highly variable from year to year, with interquartile ranges of 3.4 mm in average daily precipitation, but October is the wettest month (5.3 mm) and August is quite dry (3.5 mm). The driest months are January (2.9 mm) and February (2.8 mm).

The monthly patterns provide some clues as to the causes of differences among the gauges. For example, RG24 (in green in Fig 2) has higher catch than its neighbors from May to November, likely due to interactions of wind and topography during this predominantly rainy season; this period contains the months in which events of ≥50 mm with a south wind are most common [24]. Since RG24 is located in a concavity just north of a ridge, it could be in a zone of precipitation convergence. It is also possible that wind dynamics could be affected by the presence of leaves from May to October, even though clearings are maintained around the gauges to prevent canopy interference.

In contrast, RG3 (in red in Fig 2) is located just south of a ridge, and it collects less precipitation than its neighbors in November, December, and January, especially when collecting snow [24]. This gauge may lie in a region of flow separation during winds from the northwest in winter [36] or it may experience higher local wind speeds, which could explain low catch.

Finally, RG10 (in yellow in Fig 2) is high from October to May, when stratiform precipitation is more prevalent than convective events [24]. This gauge is located in a midslope position and it is not clear why it differs from its neighbors.

We hoped that inspection of these seasonal patterns would inform decisions as to whether to retain these unusual gauges. In the end, because the differences were quite small, other factors (described below) proved more important in deciding which gauges to retain.

### Simulating single gauge removal

The removal of a single gauge has no influence on estimates of precipitation in catchments that are widely separated from it (Table 2; Fig 1). A zero in the table indicates that removing that gauge does not influence precipitation estimates in the corresponding catchment. Omitting a gauge from the south-facing catchments does not affect calculated precipitation inputs to the north-facing catchments and vice versa. For this reason, in subsequent analyses, we analyzed the two groups of gauges and catchments independently.

**Table 2:** Percent change in aerially weighted precipitation estimates by catchment (W1 to W9) incurred by removing one gauge at a time, using IDW for spatial interpolation among gauges.

	W1	W2	W3	W4	W5	W6	W7	W8	W9
RG01	0.12	0.04	0	0.03	0	0	0	0	0
RG02	-1.6	-1.3	-0.1	-0.1	0	0	0	0	0
RG03	2.33	2.22	0.22	0.07	0	0	0	0	0
RG04	0	0.31	0.37	0	0	0	0	0	0
RG05	0	0	-0.4	0	0	0	0	0	0
RG06	0	0	0	-0.4	0.16	0	0	0	0
RG07	0	0	0	0.16	0.37	0.03	0	0	0
RG08	0	0	0	-0.1	0	0	0	0	0
RG09	0	0	0	0	0.05	0.62	0	0	0
RG10	0	0	0	0	-0.6	-1.2	0	0	0
RG11	0	0	0	0	0	0.32	0	0	0
RG12	0	0	0	0	0	0	0.12	0	0
RG13	0	0	0	0	0	0	0.26	0	0
RG14	0	0	0	0	0	0	-0.4	0	0
RG15	0	0	0	0	0	0	0.04	-0.1	0
RG17	0	0	0	0	0	0	-0.1	0	0
RG19	0	0	0	0	0	0	0.21	0.24	0.38
RG20	0	0	0	0	0	0	0	0	0
RG21	0	0	0	0	0	0	0	0.3	0
RG23	0	0	0	0	0	0	0	-0.1	0.32
RG24	0	0	0	0	0	0	0	0	-1.8
RG25	0	0	0	0	0	0	0	0	1.1

On the south-facing slope, there were three gauges that had an effect greater than 1% on estimates of long-term average catchment inputs (Table 2). Since RG10 is the wettest, removing it reduces precipitation estimates to the catchments it is in or near. RG3 is the driest, and removing it causes precipitation estimates near it to rise. RG2 is surrounded on three sides by drier gauges, and thus removing it has a large impact on two of the catchments. On the north-facing slope, removing RG24, which is the wettest of all the gauges, and RG25, which is the driest, caused deviations greater than 1% on estimated precipitation inputs to W9, the catchment they are in. The other gauges in the north-facing catchments are so similar that removing any of them had <0.4% effect on any of the catchment estimates of long-term average precipitation.

### Multi-gauge reduction

**Fig 3:**
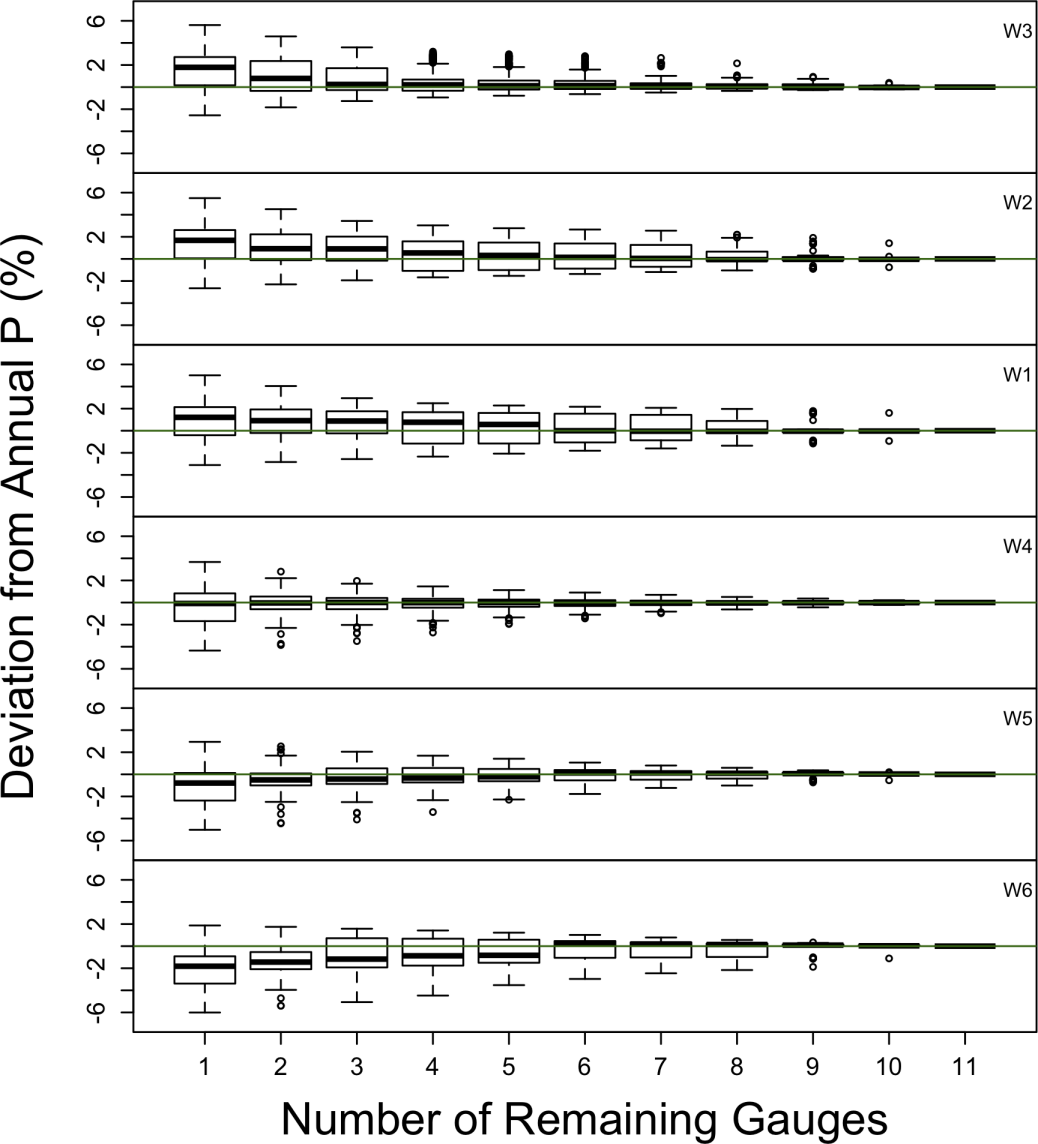
The effect of removing gauges for each of the six south-facing catchments is shown as the absolute value of the deviation of annual precipitation estimates for all possible combination of gauges. The results are shown as a function of the number of gauges remaining. The deviation from annual precipitation is based on the IDW method. The green line indicates zero deviation.

**Fig 4:**
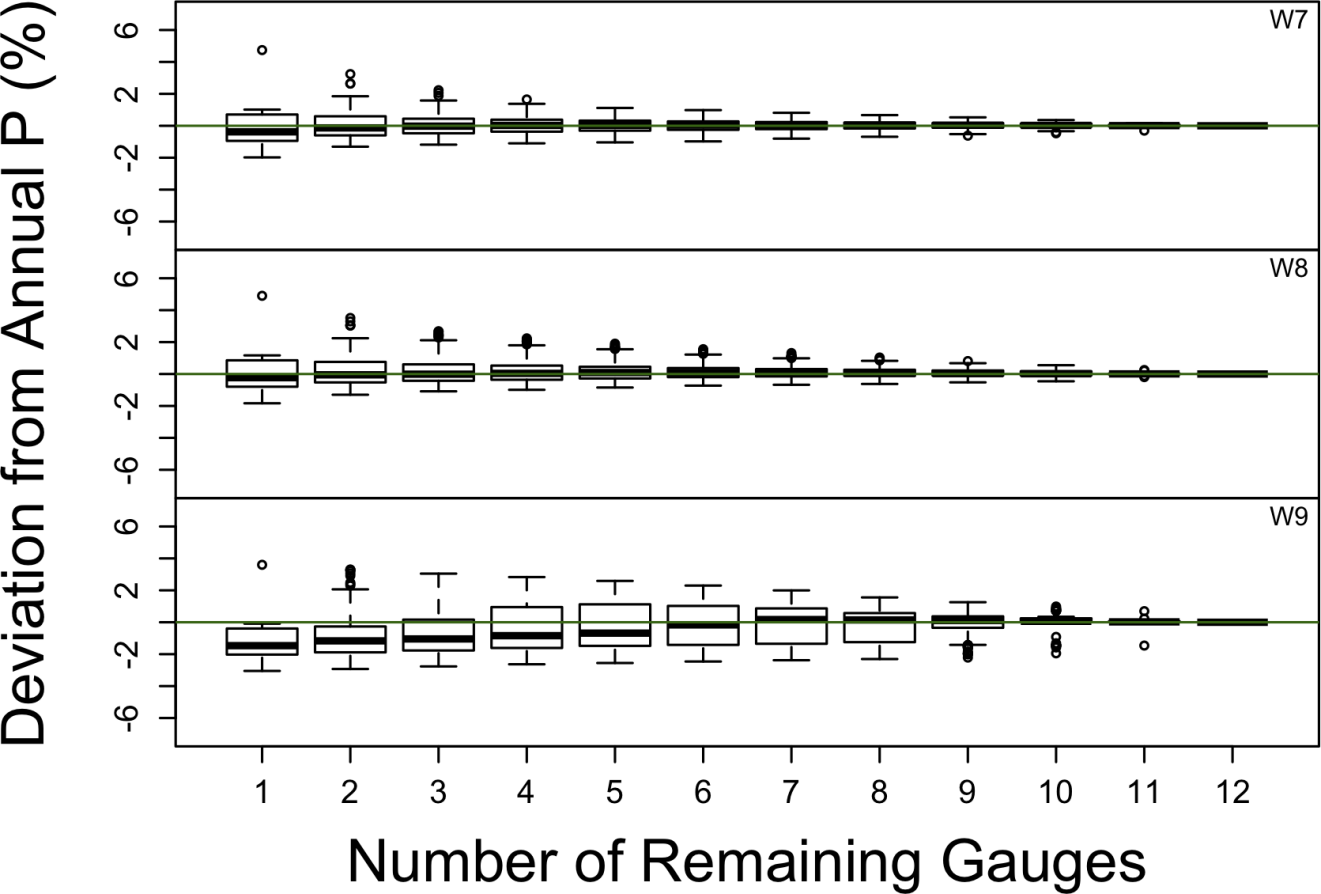
The effect of removing gauges for each of the three north-facing catchments is shown as the absolute value of the deviation of annual precipitation estimates for all possible combination of gauges. The results are shown as a function of the number of gauges remaining. The deviation from annual precipitation is based on the IDW method. The green line indicates zero deviation.

In general, scenarios that removed more gauges deviated more from the estimate based on all the gauges (Figs 3 and 4). Deviations of as much as 6.0% for individual scenarios were observed across the nine catchments, but 95% of the scenarios deviated by <2% and 74% deviated by <1%. Long-term average precipitation at the catchment scale is not very sensitive to gauge removal at Hubbard Brook.

The catchments that showed the greatest variation in precipitation estimates were those most influenced by gauges that differed from the average. Specifically, on the south-facing catchments, W1 and W2 show more sensitivity to gauge removal than W3 because RG3 accounts for a large fraction of their area, and RG3 is the driest of all the gauges in the study (Table 1). The deviations from the estimates based on the full set of gauges tended to be positive, as removing RG3 results in estimates of greater precipitation. Conversely, W6 has deviations that tend to be negative, as it is strongly influenced by RG10, which is wetter than the other gauges on the south-facing slopes. Similarly, for the north-facing catchments, W9 shows larger deviations than W7 or W8, because of the influence of RG24, the wettest in the study.

**Fig 5:**
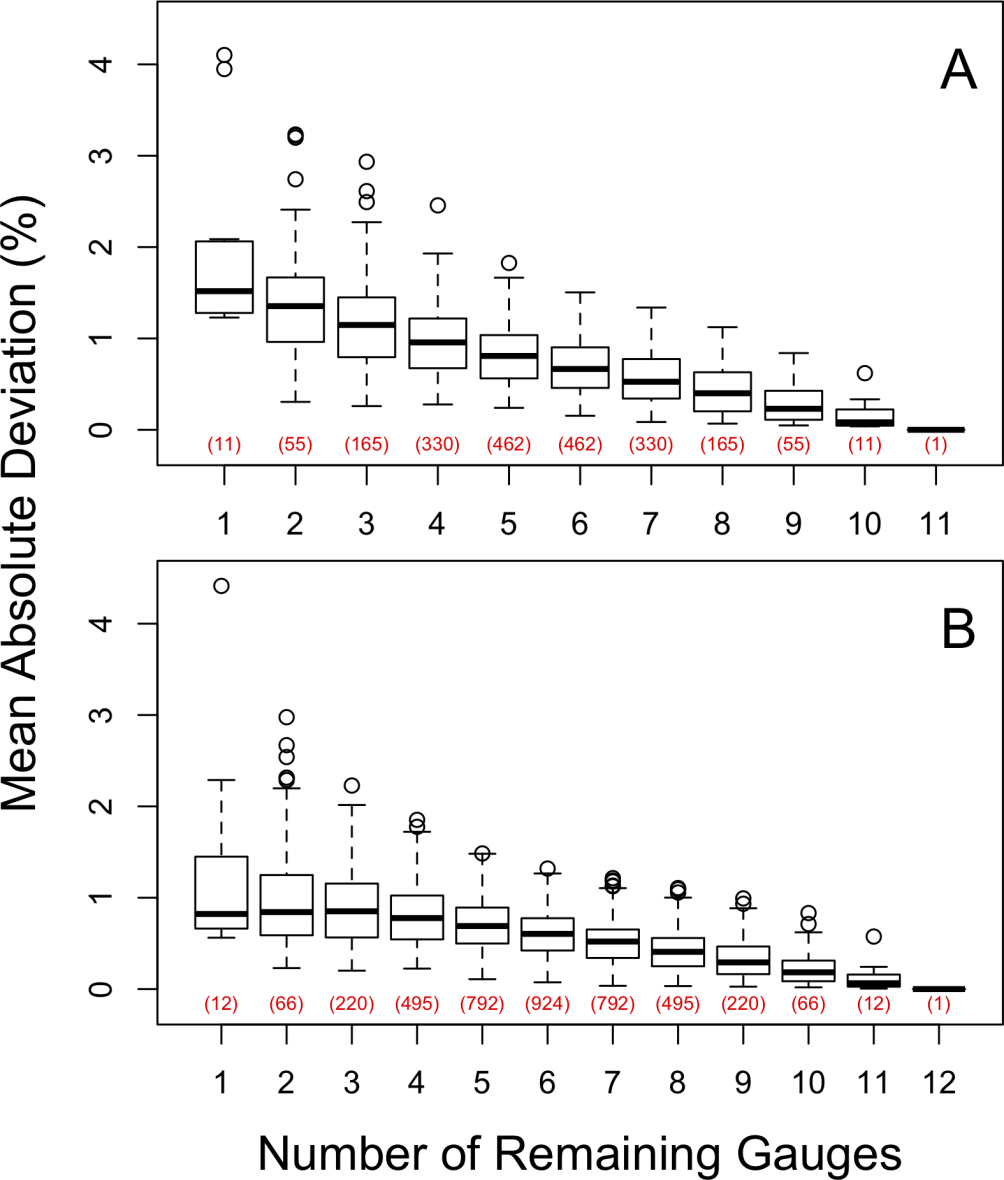
The impact of each reduction scenario (Figs 3 and 4) was summarized across the six south-facing catchments (A) and three north-facing catchments (B) by calculating the mean absolute deviation across the catchments. Red numbers in parentheses indicate the number of unique scenarios for each number of remaining gauges.

When removing gauges from a network, the decision about which gauges to remove is not made independently for each catchment, as they share gauges. Thus, we calculated the mean deviation for each scenario, using the absolute values of the deviations for all the catchments, separately for the south and north slopes (Fig 5). The median deviation for the south-facing catchments increased nearly linearly from 0 to 1.5% as gauges decreased from 11 to 1. The median deviation on the north face reached an asymptote of 0.8% at 3 remaining gauges as gauges decreased from 12 to 1.

### Impact of interpolation method

We wanted to know whether our results were sensitive to the method of interpolating precipitation among the gauges as has been observed elsewhere [37]. Differences in the long-term precipitation estimates between IDW and the Theissen approaches were 0.5% or less, for the south-facing catchments. The differences were systematic, with IDW giving drier estimates at the west end and wetter at the east (from west to east, the percent change from IDW to Theissen was W6: 0.39; W5: 0.26; W4: 0.18, W1: -0.38; W2: -0.50; W3: -0.48), consistent with RG10 being exceptionally wet at the west. For the north-facing catchments, the differences were also tiny: W7: -0.05; W8: -0.25; W9: 0.39%.

**Fig 6:**
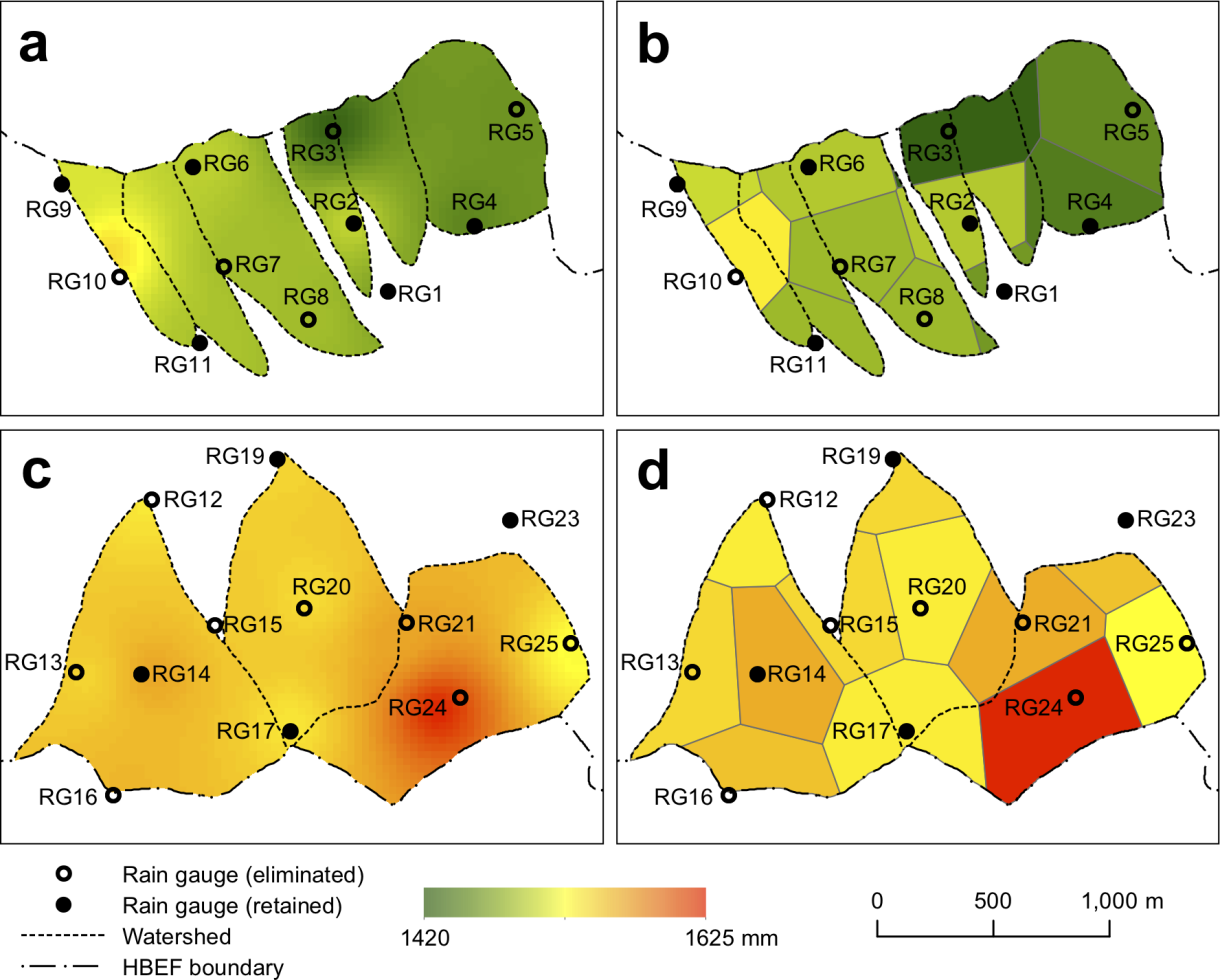
Spatial interpolation of precipitation for south-facing (a,b) and north-facing (c,d) catchments using the inverse distance weighting (a,c) and Thiessen polygon (b,d) methods. Open circles indicate gauges that were eliminated and closed circles indicate gauges that were retained.

The catchment-scale precipitation estimates used to compute the effect of reducing the number of gauges (Figs 3 and 4) used IDW to interpolate precipitation among the gauges. We also conducted this exercise using the Thiessen polygon approach to interpolation (Fig 6), with very similar results (S1 Fig is very similar to Fig 5A). The two approaches differed by a maximum of ~1%, with 90% of the scenarios differing by less than 0.5% (S2 Fig), based on long-term average precipitation. A broader suite of interpolation methods may have demonstrated more sensitivity to interpolation approach [37], particularly with daily precipitation [15].

### Impact of time scale

**Fig 7:**
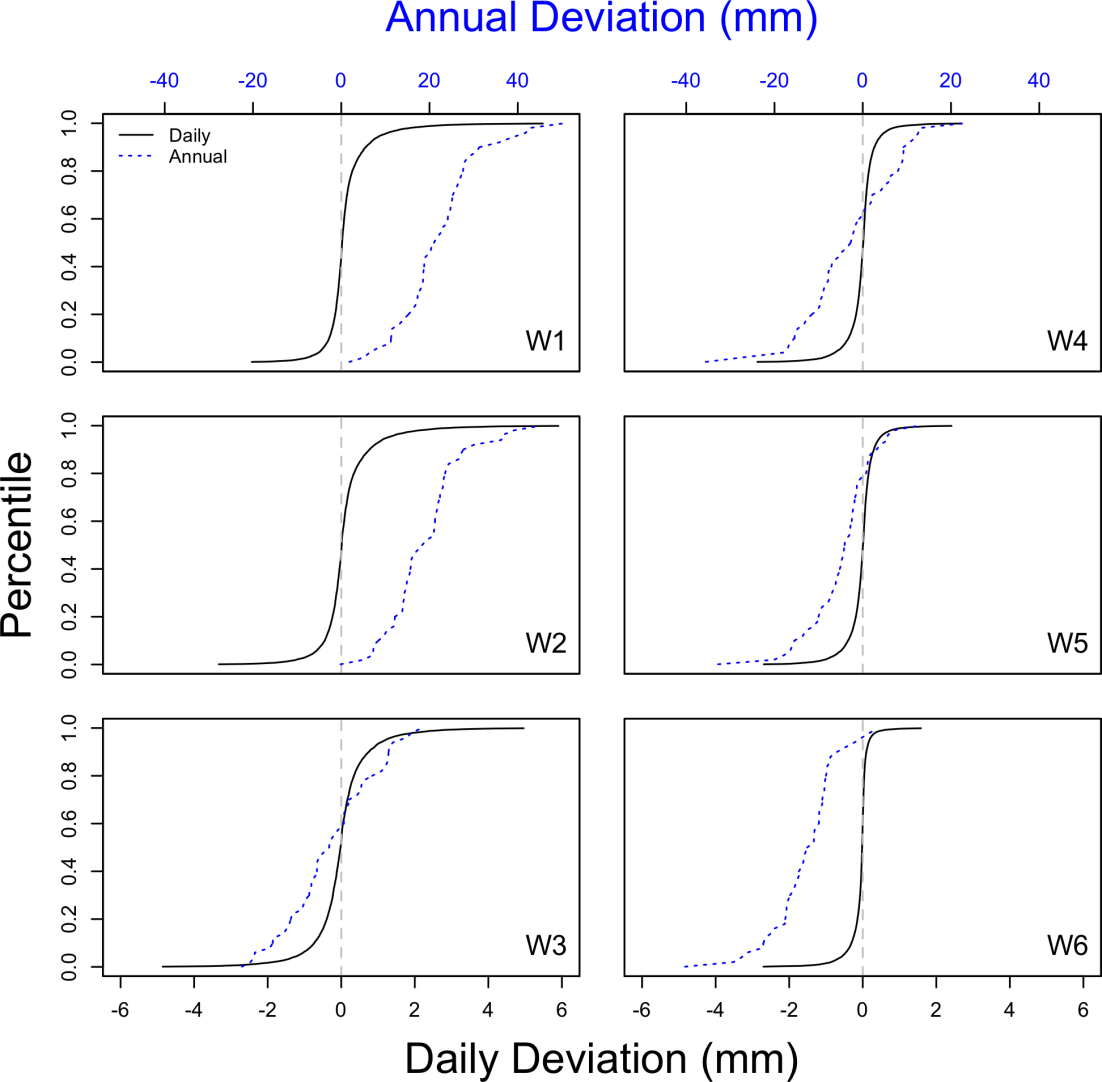
Cumulative frequency distributions of the catchment-scale precipitation deviations between estimates based on the reduced 6- gauge network (1, 2, 4, 6, 9, 11) for the south-facing catchments and estimates based on the full (12-gauge) network. The black line shows daily deviations and the blue line shows annual deviations. Only days with at least one gauge reporting precipitation were included in the daily deviation calculation. We used the precipitation record from 1964 to 2014 (the common time period when all gauges were in operation). The scale for annual deviations is at the top and for daily deviations is at the bottom. Note that the direction of skewed daily deviations, most visible in the heavy tail, corresponds with the direction of bias in the annual deviations.

The analyses of gauge removal scenarios presented above were based on the long-term average precipitation. Because the long-term average precipitation is similar across gauges (Table 1), the effects of reduced sampling were small at that time scale. To evaluate the potential loss of information at shorter time scales, we compared the distribution of deviations at the daily, monthly, and annual time scales for the south-facing catchments (Fig 7). The daily residuals were small, averaging from 0.01 to 0.1 mm, depending on the catchment, with extreme deviations rarely exceeding 5 mm. Monthly deviations averaged from 0.2 to 2 mm, with tails of the distribution nearing 10 mm (data not shown). As a percentage of the estimate using the full network, the median annual and daily deviations were similar and <1.5% for all the catchments.

The annual deviations show bias, with median values greater than zero at W1 and W2, approximately zero at W3 and W4, and below zero at W5 and W6. The biases are driven by the removal of gauges with unusually high (RG10) or low (RG3) precipitation values compared to the rest of the south facing gauges. W1 and W2 are heavily influenced by RG3, thus its removal causes a substantial positive change in the catchment-scale precipitation estimate. Conversely, W6 is heavily influenced by RG10; thus, when it is removed, the precipitation estimate markedly decreases. The annual bias direction coincides with the direction of the skew of the cumulative distribution of daily deviations. For example, W2 has a positive annual deviation, resulting from the positive skew of daily deviations (Fig 7).

### Consequences for evapotranspiration and solute flux

**Fig 8:**
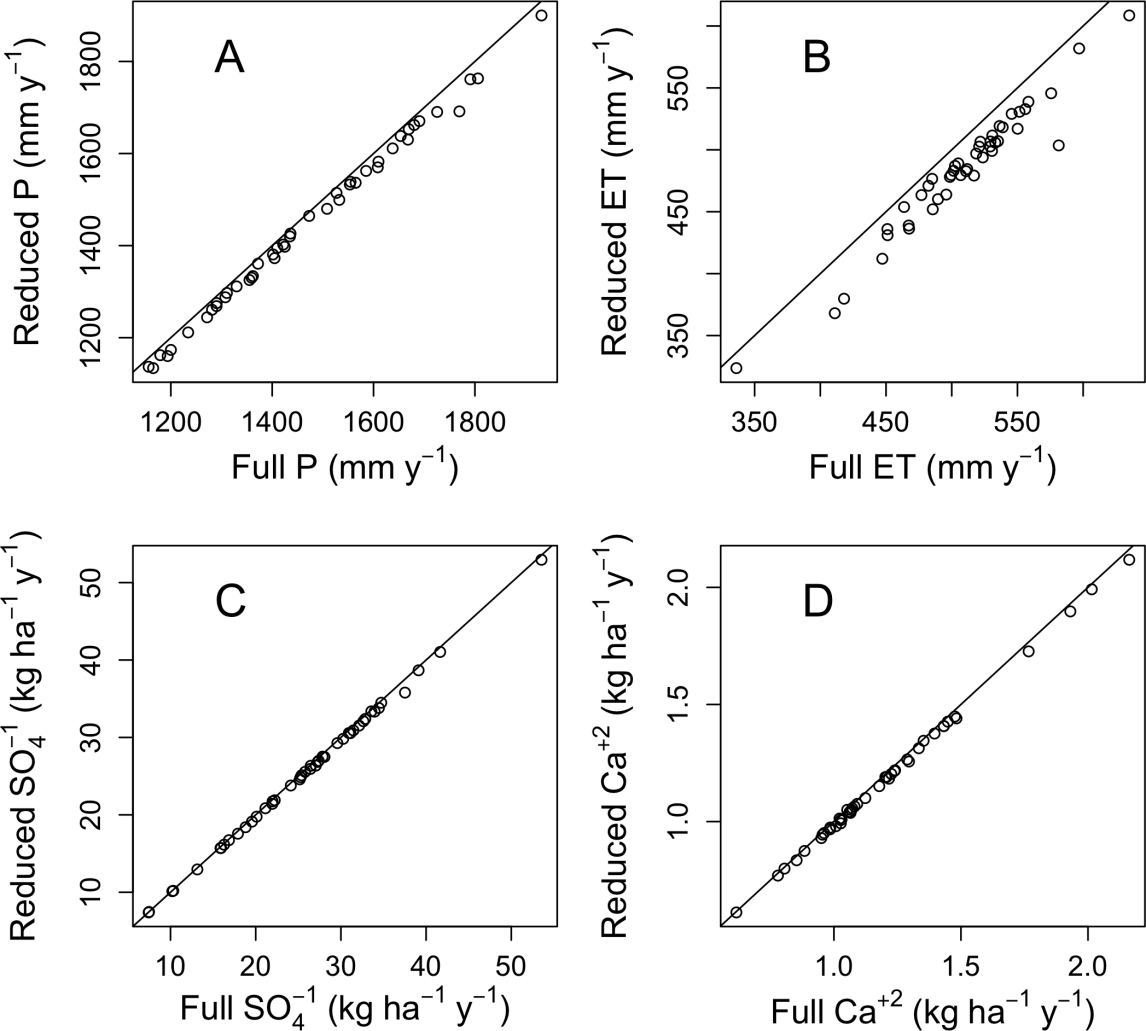
Estimates of annual precipitation (P), evapotranspiration (ET), sulfate bulk deposition, and calcium bulk deposition for W6 based on the reduced precipitation monitoring scheme compared to estimates based on the full monitoring network.

Precipitation inputs are used to estimate water budgets by subtracting streamflow to estimate evapotranspiration. They are also used to estimate inputs of solutes, by multiplying precipitation inputs by solute concentrations. We evaluated the effect of our proposed changes in precipitation monitoring for W6, the biogeochemical reference catchment. The change in precipitation estimates was small but consistently negative (Fig 8), due to the removal of RG10, which was consistently wetter than its neighbors (Fig 6), as we have already shown (Figs 3, 4 and 5). The effect of this bias on annual evapotranspiration is the same in units of mm (the median difference per year is 23 mm) but larger as a fraction of the total (1.6% of precipitation but 4.5% of evapotranspiration). For fluxes of sulfate, the dominant anion, and calcium, the dominant base cation, the changes look less important (Fig 8), because of the large interannual variation in concentrations of these solutes. This analysis is presented for the period from 1968 to 2014, which had a range of 0.5 to 3.3 mg L^-1^ in annual volume-weighted sulfate concentration and a range of 0.05 to 0.21 mg L^-1^ in calcium concentrations. A shorter record would likely have a lower range of concentrations, and the difference due to the change in precipitation monitoring scenario would be larger as a fraction of the total variation in the record.

## Discussion

### Effect of removing the wettest and driest gauges

Although the range in average precipitation across the Hubbard Brook network is small, certain gauges collect consistently high (RG10 and RG24) or low (RG3) amounts of precipitation relative to the others. These gauges are monitored in exactly the same fashion as the other gauges, and measurement error [9] is thus not likely to explain the differences. However, in some cases, local topography and weather patterns may be responsible for these patterns of high and low values, as described above. RG3, on the ridgetop on the south-facing slope, measures less precipitation than surrounding gauges in the winter months (Fig 2), suggesting that snowfall undercatch may be responsible for its low values. It is not clear why it is lower than RG6 and RG9, which are on that same ridgetop; RG3 has a higher component of conifers in the forest around it. RG24 has higher precipitation amounts than nearby gauges, especially from June to October (Fig 2); it is located in a microvalley, which may create wetter or overcatch conditions during the growing season. RG10 has no notable local vegetation or topographic characteristics that make its distinct from other gauges, however the higher precipitation there occurs during the leaf-on season. We cannot suggest why this site is wetter than its neighboring gauges.

The largest effects of proposed gauge reductions occurred when these unusual gauges were removed from the network (Table 2; Fig 5). It is impossible to know, without adding more gauges, whether these gauges represent large areas, perhaps larger than the IDW or Theissen interpolation methods credit them for, or whether catchment-scale estimates would be more accurate without them, if the areas of unusual precipitation are small. The decision to discontinue all three of them, in the end, was made due to other logistical factors (described below). Although these gauges present challenges for decisions about precipitation monitoring, they may be valuable for describing micrometeorological patterns. For example, the lower catch at RG3 occurs consistently during winter months but not summer (Fig 2). Further attention to the seasonal meteorology could help isolate mechanisms causing this reduced catch.

### Selecting a reduced monitoring scenario

Our quantitative gauge removal analysis provided information to assist in selecting a reduced monitoring scenario. For example, for the south-facing catchments, the best combination of two gauges, in terms of giving a long-term average precipitation estimate closest to the estimate based on all the gauges, was RG4 and RG10. Information of this type is valuable but not sufficient to determine the number of gauges to retain, which depends on the resources available, not just on the loss of information expected to result from the reduction.

In our case, the number of gauges we decided to retain was 10, based in part on the number of automated weight-recording gauges available. Data from these gauges are transmitted in near-real time to the headquarters building, eliminating the need to visit each gauge weekly. Remote monitoring also enables technicians to identify and respond to problems without visiting the gauges, thus reducing data loss.

Other more subjective or qualitative factors also influenced the final selection of gauges to retain. Some sites were retained because other long-term measurements were continuing at those sites, such as air temperature (RG1, 6, 14, 17, 22, 23, 24) or snow depth (RG2, 9, 17, 19), and precipitation was complementary to those measurements. Some gauges were excluded because they were more difficult to reach (RG3) or presented safety issues, especially in winter (RG24, RG25). Spatial coverage of the catchments was also a factor. There were multiple combinations of gauges that met these criteria, and the scenario finally selected was the one that produced the smallest deviation from the current monitoring scenario (Figs 3 and 4). A quantitative cost algorithm was discussed, which would estimate how long would it take to visit all the gauges depending on which ones were dropped, but this tool was not needed, in the end, to make a decision.

The final scenario we selected included six precipitation gauges on the south-facing slope (RG1, 2, 4, 6, 9, and 11), and four on the north-facing slope (RG14, 17, 19, and 23). The reduction in the time and effort required to monitor these gauges is even greater than the proportion dropped (5 of 11 for the south-facing slopes and 8 of 12 for the north-facing slopes), because the gauges most difficult to access are among those that were discontinued. Thus the benefit of making this reduction seems to far exceed the cost in loss of information, given our monitoring priorities and budget constraints.

### Strategies for evaluating monitoring networks

Our approach to evaluating the precipitation monitoring network at Hubbard Brook involved simulating all possible gauge reduction scenarios and testing the impact of these scenarios on the estimates of ecosystem fluxes that are the scientific focus at our site (Fig 8). Another possible approach is to use principal component analysis to provide information about how individual gauges contribute information about the spatio-temporal variability of precipitation within the network. We tried this approach, and found that the first principal component, which correlated with daily precipitation, described 97% of the variation without separating the gauges (S3 and S4 Figs). The second principal component separated the gauges on the north- and south-facing slopes, which our analysis treated separately, and the third component grouped gauges by spatial location, consistent with our proposed reduction scheme. Although in our case, these components explained very little of the variability in precipitation, this approach has been useful in assessing many regional networks, where spatial variability is larger and more of the principal components explain substantial variability [38–40].

Our proposed reduction in precipitation monitoring effort will have very little effect on estimates of precipitation volume. The difference in the long-term estimates of precipitation based on the reduced scheme averages 1.1% for the south-facing catchments and 0.9% for the north-facing catchments. The difference is only slighter greater for annual water budgets (Fig 8), which have been the focus of precipitation monitoring at Hubbard Brook [7]. The uncertainty associated with daily precipitation (Fig 7) is slightly greater due to the spatial heterogeneity of storms.

Even though we are reducing our network, we see great value in spatially dense precipitation monitoring. A denser precipitation network would provide better information for understanding processes for individual storm events. For example, describing the spatial variability of soil responses (e.g., soil wetting, soil redox dynamics) to storm events would require detailed characterization of spatial precipitation patterns for each storm. Given the small elevational range and relatively low topographic complexity at Hubbard Brook, even our reduced network will provide adequate precision to address some storm-scale research questions. However, characterizing heterogeneity at fine spatial or temporal scales might benefit from greater sampling intensity.

While more precipitation gauges provide more information, the value of the information gained has to be weighed against the costs associated with the additional gauges. These costs include the labor associated with visiting the gauge, maintenance of the gauge and clearing that surrounds it, and quality control and management of the data. Although even more gauges could have been eliminated without a substantial increase in error, there are risks associated with having too few gauges. At Hubbard Brook, gaps in precipitation data are rare, but data can be lost for several reasons, mostly due to equipment failure and technician error [9]. Having multiple gauges makes it possible to fill gaps in the precipitation record based on long-term relationships among gauges. Multiple gauges could be helpful in identifying measurement errors if they are co-located [41], because precipitation can differ even over short distances.

### Addressing the issue of methodological change

Methodological change is inevitable with long-term data, and it is critical to correct the data or clearly document the transition to ensure that trends are not misrepresented and the data are correctly interpreted [42,43]. In our case study there are two examples of methodological change: the transition from Thiessen weighting to IDW and the transition from the full suite of precipitation gauges to the reduced scenario. These changes could result in small differences in the long-term records of precipitation and variables dependent on precipitation (i.e., evapotranspiration and solute deposition).

One option is to make corrections to the historical record to bring it into agreement with the record going forward. In our case, we could recalculate inputs to catchments based on IDW, going back to 1955. Although there is always reluctance to change previously published values, in cases like this when an alternative method is better than the method used in the past, there is a good argument for recalculating the entire record with the new method. Another option is to provide the revised data set as a second version of the original data set.

In the case of reducing the number of precipitation gauges, the past method using the full suite of gauges provided more information than the new method, which uses fewer gauges. It would be possible to report the historical data based on fewer gauges, but this would ignore the information provided by having had a denser network for many years. An alternative is to use the data from the full suite of gauges to inform the precipitation estimates based on the remaining gauges going forward. For example, at the Coweeta Hydrologic Laboratory in North Carolina, USA, a precipitation network consisting of 69 gauges was maintained from 1951 to 1958 [20,44]. The network was then reduced to 9 gauges and the interpolation procedure was based on maps derived using the discontinued gauges.

In summary, the options for dealing with changes in methods that affect the results are to (1) make corrections to the historical record consistent with the future record, (2) make corrections to the future record based on the past record, (3) report the historical record and explain the step function at the time the change was made. If the historical record is changed, we recommend archiving the original record and making it available.

## Conclusion

We used a quantitative analysis to determine how the elimination of gauges from a network would impact estimates of precipitation, evapotranspiration, and solute deposition at small catchments at the Hubbard Brook Experimental Forest. Ultimately, the decision about gauges to retain was also heavily influenced by practical considerations, such as the accessibility of individual gauges and cost for adding and maintaining automated gauges. This hybrid approach to decision-making was useful in this case because the practical considerations constrained the number of scenarios, and the quantitative analysis made it possible for us to select among the remaining candidates and select a scenario with satisfactory precipitation estimates. This case study illustrates how quantitative and qualitative information can be used to revise monitoring networks and improve efficiency while minimizing loss of information.

## Supporting information

S1 Fig Distribution of mean absolute error for all gauge-reduction scenarios, grouped by the number of remaining gauges on the south-facing slope. This is the same as Fig 5A, except it uses Thiessen polygon interpolation.
(PDF)

S2 Fig The distribution of differences between scenarios using IDW (Fig 5A) and Thiessen polygons (S1 Fig) for the south facing slope. (PDF)

S3 Fig Biplot of principal components 1 and 2 with the vector for each precipitation gauge. (PDF)

S4 Fig Biplot of principal components 2 and 3 with the vector for each precipitation gauge. (PDF)

S1 Text Principal component analysis. (PDF)
