# Supplementary figures and images for: Downsizing a long-term precipitation network: Using a quantitative approach to inform difficult decisions
Source: PLoS One. 2018 May 7;13(5):e0195966. doi: 10.1371/journal.pone.0195966 (PMC5937762; doi:10.1371/journal.pone.0195966)

S1 Figure

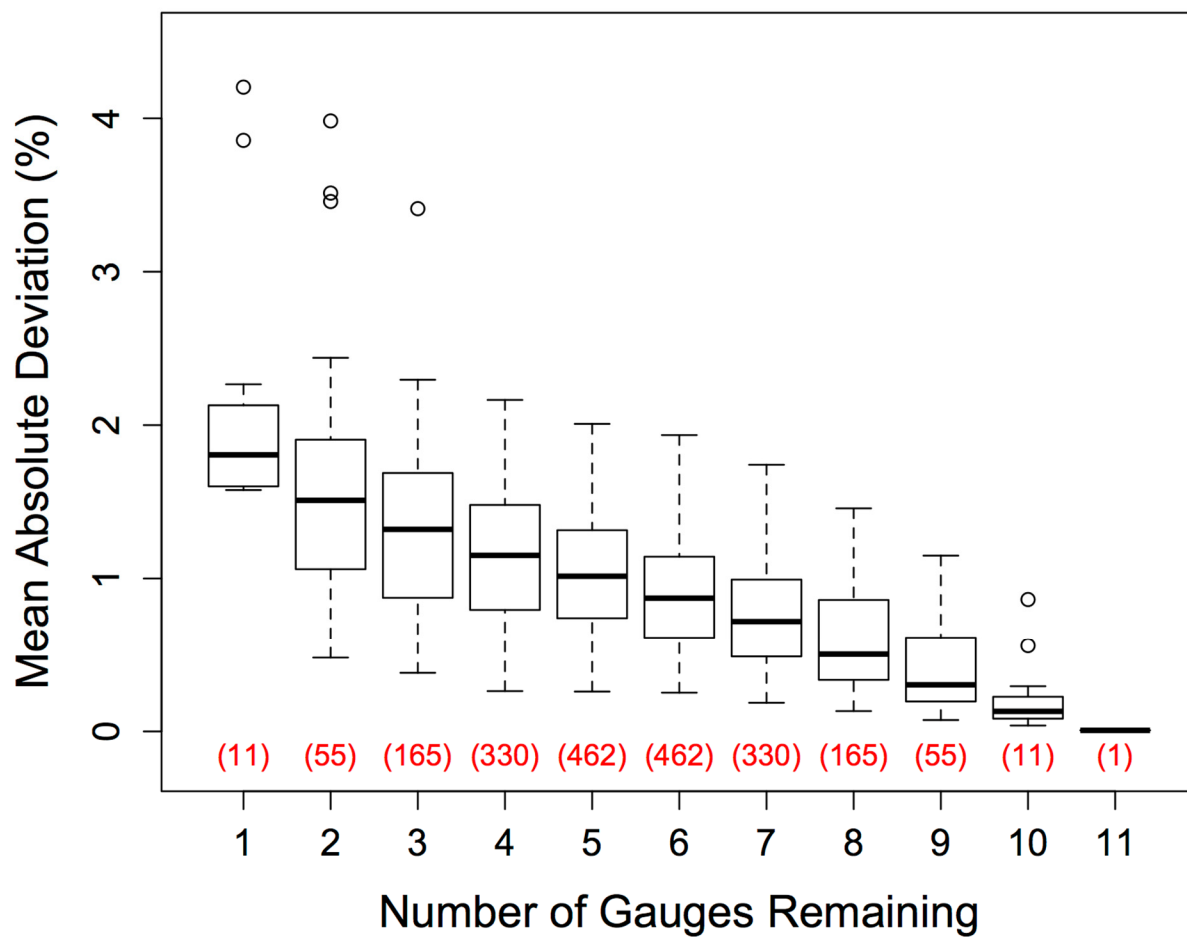

Supplement: S1 Fig — This is the same as Fig 5A, except it uses Thiessen polygon interpolation. (PDF) [file pone.0195966.s001.pdf]

S2 Fig

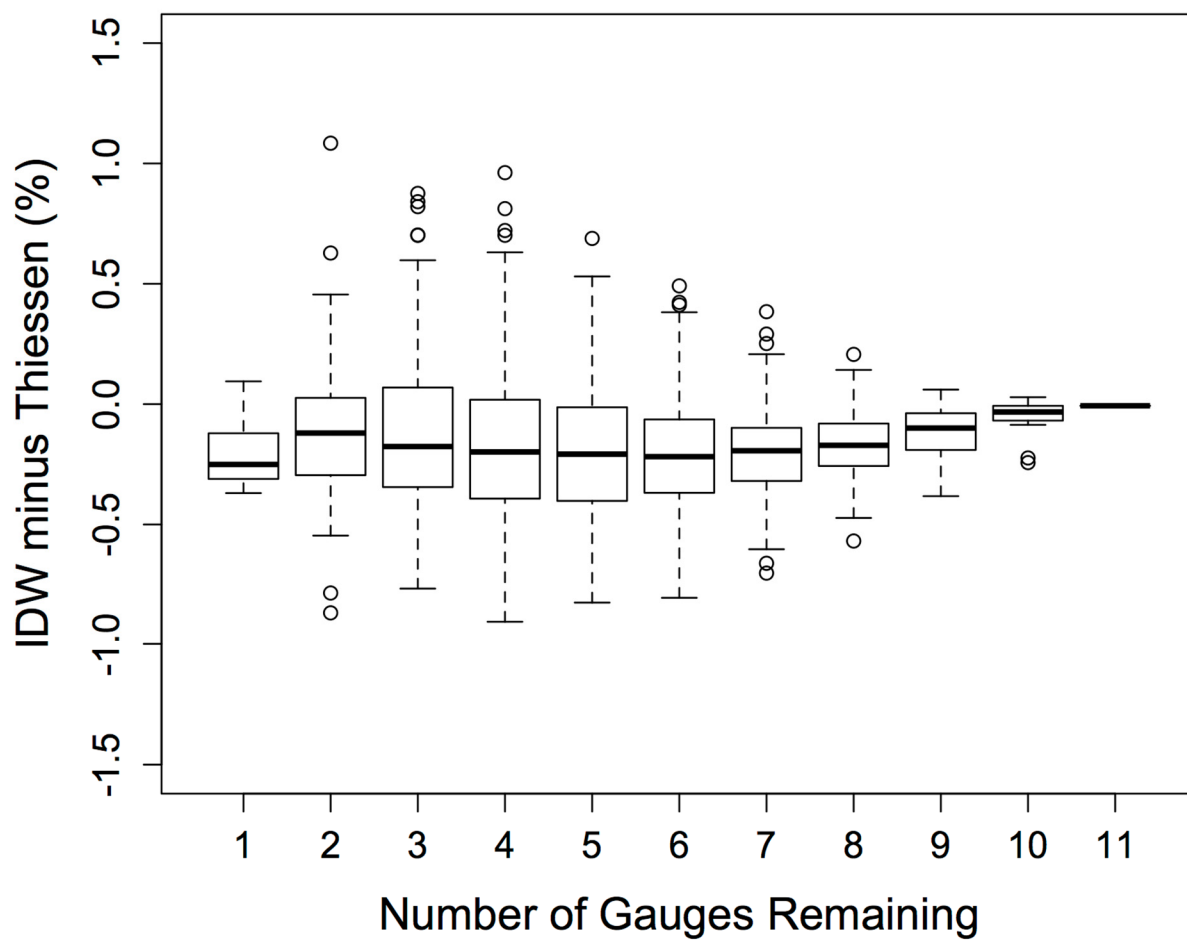

Supplement: S2 Fig — (PDF) [file pone.0195966.s002.pdf]

S3 Fig

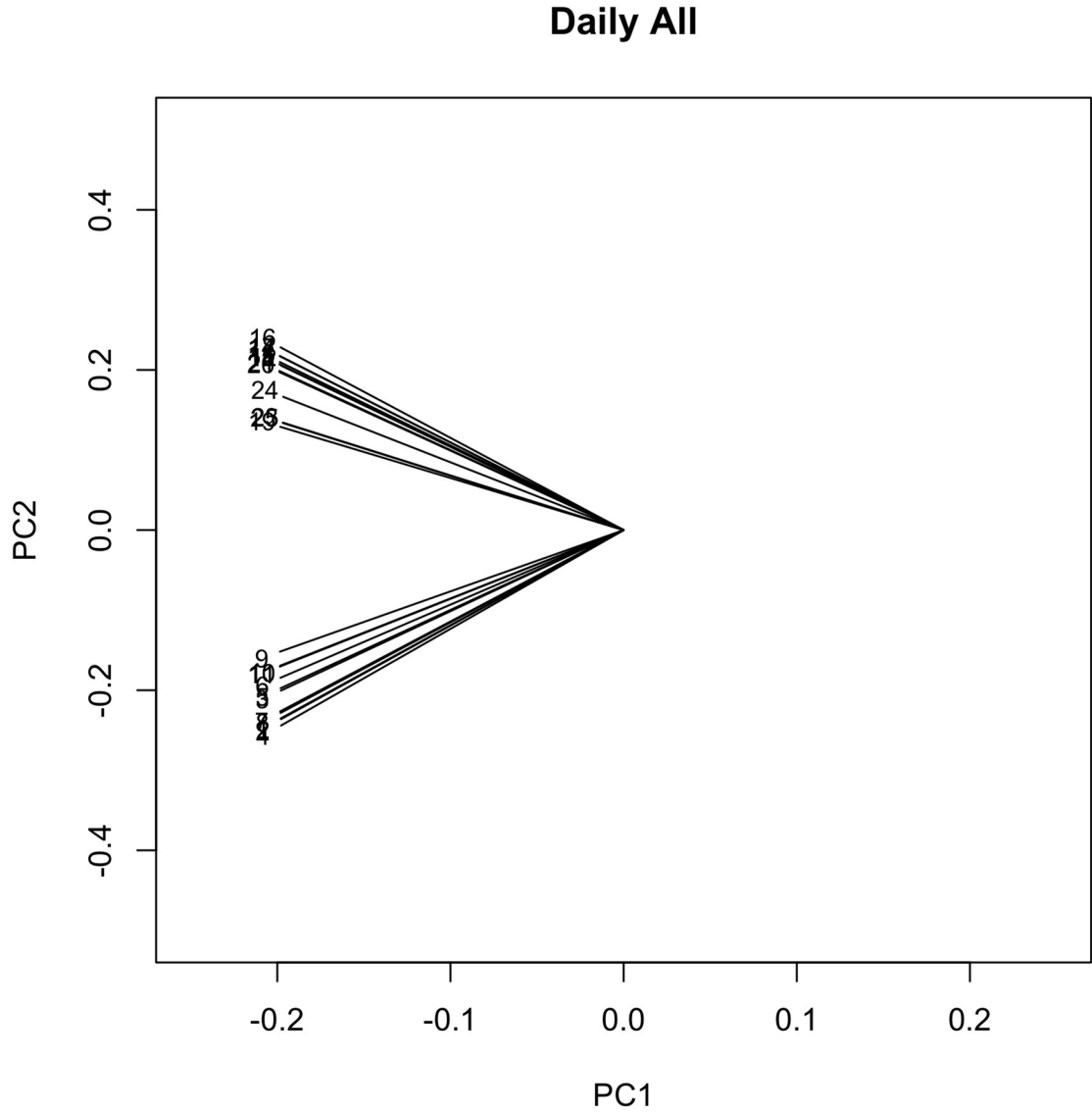

Supplement: S3 Fig — (PDF) [file pone.0195966.s003.pdf]

S4 Fig

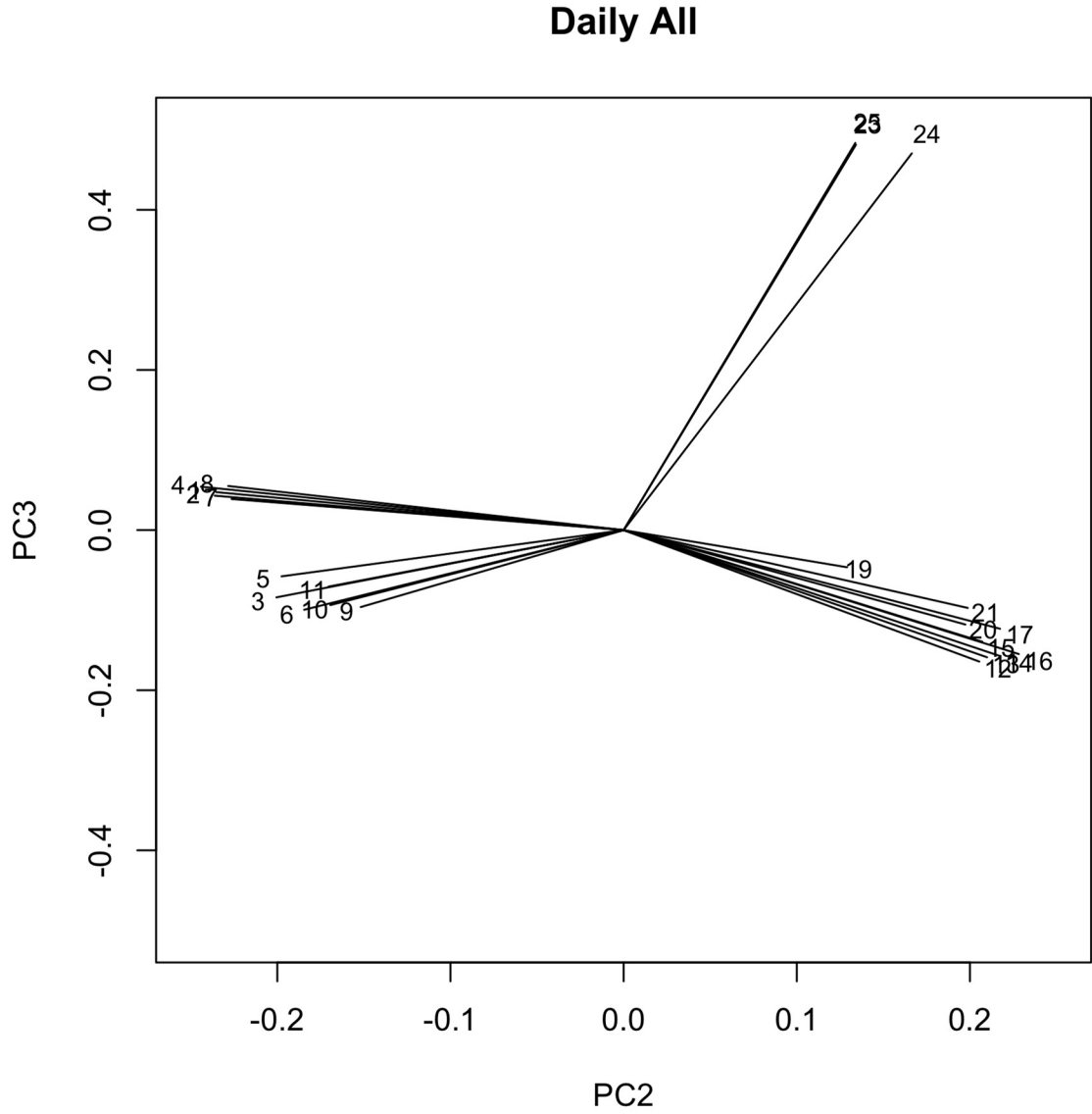

Supplement: S4 Fig — (PDF) [file pone.0195966.s004.pdf]
