# Supplementary material for: Downsizing a long-term precipitation network: Using a quantitative approach to inform difficult decisions
Source: PLoS One. 2018 May 7;13(5):e0195966. doi: 10.1371/journal.pone.0195966 (PMC5937762; doi:10.1371/journal.pone.0195966)
Supplement: S1 Text — (PDF) [file pone.0195966.s005.pdf]

S1 Text. Principal Component Analysis.

We conducted a principal component analysis on the daily precipitation values from the 23 gauges between January 1998 and December 2014 using the princomp base function in R. The results showed that principal component 1 (PC1) explains 97.4% of the variance, principal component 2 (PC2) explains 1.3% of the variance, and principal component 3 (PC3) explains 0.5% of the variance. The biplot of P1 and PC2 shows that the gauges separate by topographic aspect (S3 Fig). The biplot of PC2 and PC3 shows separation of gauges into two geographic groups within each aspect group (S4 Fig). Our gauge reduction scenario retains at least one gauge from each of the four groups shown in S4 Fig.
